# PD-1/PD-L1 in Cancer: Pathophysiological, Diagnostic and Therapeutic Aspects

**DOI:** 10.3390/ijms22105123

**Published:** 2021-05-12

**Authors:** Enrico Munari, Francesca R. Mariotti, Linda Quatrini, Pietro Bertoglio, Nicola Tumino, Paola Vacca, Albino Eccher, Francesco Ciompi, Matteo Brunelli, Guido Martignoni, Giuseppe Bogina, Lorenzo Moretta

**Affiliations:** 1 Pathology Unit, Department of Molecular and Translational Medicine, University of Brescia, 25100 Brescia, Italy; enrico.munari@unibs.it; 2 Immunology Area, Bambino Gesù Children’s Hospital, IRCCS, 00146 Rome, Italy; fromana.mariotti@opbg.net (F.R.M.); quatrini.linda@opbg.net (L.Q.); tumino.nicola@opbg.net (N.T.); vacca.paola@opbg.net (P.V.); 3 Division of Thoracic Surgery, IRCCS Maggiore Teaching Hospital and Sant’Orsola University Hospital, 40133 Bologna, Italy; pieberto@hotmail.com; 4 Pathology Unit, University and Hospital Trust of Verona, 37134 Verona, Italy; albino.eccher@aovr.veneto.it; 5 Computational Pathology Group, Department of Pathology, Radboud University Medical Center, 6543 SH Nijmegen, The Netherlands; f.ciompi@gmail.com; 6 Department of Diagnostics and Public Health, University of Verona, 37134 Verona, Italy; matteo.brunelli@univr.it (M.B.); guido.martignoni@univr.it (G.M.); 7 Pathology Unit, Pederzoli Hospital, 37019 Peschiera del Garda, Italy; 8 Pathology Unit, IRCCS Sacro Cuore Don Calabria, 37024 Negrar di Valpolicella, Italy; giuseppe.bogina@sacrocuore.it

**Keywords:** NK cells, PD-1, PD-L1, glucocorticoids, immunotherapy, cancer

## Abstract

Immune evasion is a key strategy adopted by tumor cells to escape the immune system while promoting their survival and metastatic spreading. Indeed, several mechanisms have been developed by tumors to inhibit immune responses. PD-1 is a cell surface inhibitory receptor, which plays a major physiological role in the maintenance of peripheral tolerance. In pathological conditions, activation of the PD-1/PD-Ls signaling pathway may block immune cell activation, a mechanism exploited by tumor cells to evade the antitumor immune control. Targeting the PD-1/PD-L1 axis has represented a major breakthrough in cancer treatment. Indeed, the success of PD-1 blockade immunotherapies represents an unprecedented success in the treatment of different cancer types. To improve the therapeutic efficacy, a deeper understanding of the mechanisms regulating PD-1 expression and signaling in the tumor context is required. We provide an overview of the current knowledge of PD-1 expression on both tumor-infiltrating T and NK cells, summarizing the recent evidence on the stimuli regulating its expression. We also highlight perspectives and limitations of the role of PD-L1 expression as a predictive marker, discuss well-established and novel potential approaches to improve patient selection and clinical outcome and summarize current indications for anti-PD1/PD-L1 immunotherapy.

## 1. Introduction

Programmed cell death protein 1 (PD-1) was initially discovered as an apoptosis-associated gene during T cell thymic selection. It was shown to be involved in the regulation of the immune response and is considered one of the most important inhibitory checkpoints [1,2,3,4]. PD-1 belongs to the CD28/cytotoxic T-lymphocyte-associated protein 4 (CTLA-4) subfamily of the immunoglobulin (Ig) superfamily [5] and is expressed on T, B, myeloid and Natural Killer (NK) cells [6,7]. PD-1 specifically interacts with programmed death ligand 1 (PD-L1) and programmed death ligand 2 (PD-L2). Of note, PD-L1 is expressed in both hematopoietic and non-hematopoietic cells and on antigen-presenting cells (APC), while PD-L2 is found, upon cell activation, on macrophages and dendritic cells [8,9,10]. The binding of PD-1 to its ligands impairs T cell receptor (TCR) signaling T cell activation.

The level of PD-1 expression on T cells is related to the strength of TCR signaling. Its physiological role is to counteract excessive T cell activation while it returns to basal levels after the antigen has been cleared. However, the persistence of antigen stimulation, occurring in both chronic viral infection and cancer, may determine a constitutive PD-1 cell surface expression leading to the inhibition of immune response and impaired T cell function. In this context, due both to the tumor immunosuppressive environment and to prolonged exposure to tumor antigens, high PD-1 expression may be detected on tumor-infiltrating lymphocytes (TILs), which is associated with defects in immune cell function and the expression of other inhibitory receptors [11,12]. Therefore, it is conceivable that this mechanism has been “stolen” by tumor cells to favor peripheral tolerance and escape the antitumor immune response. In this context, the expression of PD-1 ligands has been frequently detected in different tumors, such as neuroblastoma (NB), melanoma, lung and gastric cancers. It appears that the stimuli present in the tumor microenvironment (TME) promote the expression of these PD-1 ligands [13,14,15]. In vivo studies revealed that the inhibition of the PD-1/PD-Ls axis could restore immune cell function, thus providing a promising strategy for immunotherapy [16]. Indeed, the use of anti-PD-1 and anti-PD-L1 monoclonal antibodies (mAbs), in view of particularly encouraging results in patients with lung or melanoma, have been approved by the Food and Drug Administration (FDA) [17,18] (see paragraph 5.0). In this review, we highlight the recent findings on PD-1 expression on both NK and T cells in the tumor context. In addition, we provide an overview of the different diagnostic approaches to improve patient selection and the therapeutic strategies to target the PD-1/PD-L1 axis alone or in combination therapies in several adult and pediatric cancers.

## 2. PD-1 Expression in Antitumor Effector T and NK Cells

Investigating PD-1 expression in TILs is essential in order to better identify mechanisms adopted by tumor cells to evade the control by the immune system and to develop more efficient therapies.

Of note, immune infiltrates may contain not only T but also NK cells. T cells, after encountering tumor antigens in tumor-draining lymph nodes, acquire effector function and migrate to the tumor site to promote tumor eradication [19]. However, once at the tumor site, CD8^+^ effector T cells have to overcome the inhibitory signals present in the TME. A number of studies demonstrated that CD8^+^ T cells lose their effector function during tumor progression, an effect mainly related to inhibitory checkpoint expression [20,21,22]. Of note, both CD4^+^ PD-1^+^ and CD8^+^ PD-1^+^ T cells have been detected in different tumors, including head and neck, gastric, breast and lung cancers, melanoma and hepatocellular carcinoma (Table 1) [11,12,23,24,25,26]. Kumagai and colleagues analyzed the CD8^+^ PD-1^+^ subpopulation in non-small cell lung cancer (NSCLC), gastric cancer (GC) and malignant melanoma (MM) treated with mAb therapy. They showed that higher PD-1 expression on CD8^+^ TILs reflects the interaction with tumor antigens and can be considered a predictive biomarker for delivering therapeutic antibodies able to disrupt the PD-1/PD-L1 interaction [27]. These authors also detected high numbers of PD-1^+^ CD8^+^ T cells in the TME of responders as compared to non-responder patients. In particular, in both murine models and human samples, a lower expression of PD-1 on regulatory T cells (Treg) and a higher PD-1 expression on CD8^+^ T cells correlate with a favorable antitumor efficacy of mAb treatment. In NSCLC, the frequency of PD-1 CD8^+^ TILs located in proximity to neoplastic cells is inversely correlated with NSCLC clinical staging [28]. PD-1 expression has also been detected on CD4^+^ T cells in the TME of Hodgkin lymphomas and follicular lymphomas, suggesting their role in the PD-1-induced blockade of antitumor immunity [29,30]. Li et al. analyzed the expression of inhibitory checkpoints in peripheral blood (PB) T cells in comparison to TILs in a cohort of primary and treatment-naïve patients with different tumors and found a higher PD-1^+^ cell frequency in TILs as compared to T cells from PB [31]. These results are in line with previous studies in lung, liver, esophageal and breast cancers showing that the percentage of PD-1^+^ CD8^+^ cells present in PB and at the tumor site may be considered as a prognostic marker for response to mAb immunotherapy [32,33,34,35].

Of note, clear evidence of PD-1 expression on NK cells has recently emerged (Table 1). Under physiological conditions, different from T cells, PD-1 expression in NK cells is restricted to a subset of fully mature, circulating cells in cytomegalovirus (CMV)-seropositive, otherwise healthy individuals and is maintained stable over time [7]. Several studies revealed the presence of PD-1^+^ NK cells in the PB of patients with multiple myeloma and digestive cancers [36,37]. The presence of PD-1^+^ NK cells was also detected in CD56^dim^ and CD56^bright^ NK cell subsets in Kaposi sarcoma and Hodgkin lymphoma patients, respectively [38,39]. Similarly, in the PB of renal cell carcinoma patients, PD-1 expression was restricted to a mature, cytolytic CD56^dim^ NK subset and correlated with the stage of the disease [40]. Hsu et al. showed that in lymphoma mouse models, PD-1 is expressed on a discrete fraction of NK cells represented by the most activated and functionally responsive intratumoral NK population [41]. PD-1 engagement by PD-L1^+^ tumor cells suppresses NK cell-mediated cytotoxicity, while targeting of the PD-1/PD-L1 axis allows the reactivation of NK responses. The inhibition of NK cytotoxic function, mediated by PD-1, was also detected in PD-1^+^ NK cells identified in the peritoneal fluid of ovarian carcinoma patients, demonstrating a pivotal role of PD-1 in regulating the NK cell function [7]. The impaired expression of CD107a and release of perforin and granzyme B together with a weaker antitumor activity characterized the NK PD-1^+^ cells detected in the PB of lung cancer patients [42]. In addition, a recent study on NSCLC patients showed that tumor-infiltrating NK cells express PD-1, as well as other inhibitory checkpoints, and that their dysfunction correlates with increasing levels of membrane PD-1 expression [43]. Of note, PD-1 is expressed not only on NK cells but also on innate lymphoid cells (ILCs), a heterogeneous group of cells belonging to the lymphoid lineage that is classified, according to both the transcriptional factors required for development and their functions, in different groups (ILC1, ILC2, ILC3 and LTi-like) that mirror T cell counterparts and play a pivotal role in tissue repair and immune defense (Table 1). In particular, we demonstrated that ILC3 cells from pleural effusions of patients with primary or metastatic pleural tumors expressed functional PD-1 [44]. PD-1 expression has also been found on ILC2 and ILC3 in gastrointestinal tumors [45]. Despite the increasing information on PD-1, a better understanding of the mechanisms regulating its expression and signaling in NK and helper ILC cells in the tumor context is required in order to further improve the current therapeutic approaches aimed to unleash PD-1-dependent immune cell blockade [46]. In this context, a recent study shed some light on how the TME milieu could induce PD-1 expression on NK cells [47].

## 3. Mechanisms of PD-1 Expression

Since the interaction between PD-1 on T/NK effector cells and PD-L1 on tumor cells represents a major pathway for immune evasion, many studies have been conducted to identify the mechanisms responsible for the expression of this checkpoint. In a setting different from the tumor, it was demonstrated that endogenous glucocorticoids (GCs) in combination with the cytokines IL-15 and IL-18 induce PD-1 expression at the transcript level on murine splenic NK cells upon viral infection [48]. The requirement of GCs for the de novo PD-1 expression was also confirmed in human NK cells. Indeed, high levels of cortisol were detected in the pleural effusions from patients with lung cancer, associated with increased numbers of PD-1^+^ NK cells in the tumor microenvironment [44,47]. However, GCs alone were not sufficient, but inflammatory cytokines were also needed for PD-1 expression. Interestingly, in humans, NK cell stimulation with IL-12 was also required in addition to IL-15, IL18 and GCs. Moreover, in human NK cells, PD-1 expression was not only induced by GCs at the transcriptional level, but also at the post-transcriptional level [47]. Although in T cells GCs are not required for PD-1 expression, it was shown that they can further enhance it on both mouse and human T cells [49]. Accordingly, GCs potentiate the inhibitory effect of PD-1 on antigen-dependent T cell activation [50]. Of note, GC treatment enhances not only PD-1 but also Tim3 and Lag3 expression in CD8^+^ T lymphocytes, both in mouse and human settings, through GC receptor (GR)-mediated transactivation. The authors showed an increase in the expression of the gene encoding the GR in terminally dysfunctional tumor-infiltrating T cells, and the existence of a correlation between active GC signaling and failure to respond to the checkpoint blockade in both preclinical tumor models and melanoma patients [51]. Therefore, PD-1 induction on effector T and NK cells represents an additional mechanism of immune suppression exerted by GCs, suggesting that corticosteroid therapy may be counterproductive in combination with PD-1 immune checkpoint blockade in patients with cancer. Recently, in a retrospective study, the impact of systemic corticosteroids in combination with immune checkpoint inhibitors (ICIs) was investigated to address this issue. It was concluded that GC use for an application other than immune-related adverse effects (irAE) has a negative impact on overall survival and response to ICIs, while GC therapy for irAE does not significantly affect response to immunotherapy [52].

## 4. Applications of PD-L1 Immunohistochemistry

The selection of patients who may benefit the most from anti-PD-1/PD-L1 therapies is a challenging issue, since a relevant percentage of patients do not respond to these treatments [53,54,55]. For this reason, much effort has been made to develop biomarkers with predictive potential. The best known and most used biomarker to date is PD-L1 expression, detected by immunohistochemistry, on tumor cells and/or immune cells. Multiple assays designed to assess PD-L1 expression have been developed in parallel with different antibodies targeting the PD1/PD-L1 axis. However, not all assays have the same level of interchangeability, and analysis on the same samples can lead to different results, with important implications for patient eligibility for treatment with anti-PD1/PD-L1 antibodies.

Three of the FDA-approved assays are classified as companion diagnostics, meaning that they provide information that is essential for the safe and effective use of the corresponding drug. Specifically, these assays are Ventana SP142 for atezolizumab (anti-PD-L1) in patients with urothelial carcinoma, triple-negative breast cancer or NSCLC; Dako 28-8 for nivolumab (anti-PD1) in combination with ipilimumab (anti-CTLA-4) in patients with NSCLC and Dako 22C3 for pembrolizumab (anti-PD1) in different solid tumors [56]. The FDA has indicated Ventana SP263 and Dako 28-8 as complementary diagnostics for nivolumab in advanced NSCLC and durvalumab (anti-PD-L1) for advanced urothelial carcinoma [57,58]. This means that such tests identify subsets of patients that might respond well to the therapy, but are not prerequisites for receiving the drug.

**Figure 1:**
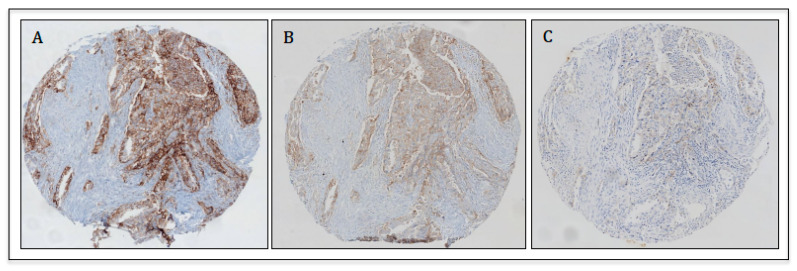
Representative image of PD-L1 expression on the same core with different assays (10× magnification): (**A**) SP263 on Ventana platform; (**B**) 22C3 on Ventana platform; (**C**) 22C3 on Dako platform.

Different commercially available assays may thus be used to make important clinical decisions; however, in clinical practice, it is challenging to make all such testing available to patients. In this regard, harmonization studies to analyze results obtained with different antibodies have been conducted [59,60,61]. Taken together, these studies suggest that three PD-L1 assays protocols (SP263, 22C3 and 28-8) have similar analytical performance and can be used interchangeably. However, our group has demonstrated that although assays 22C3 and SP263 appear to be comparable in terms of overall agreement, they show important discrepancies in identifying positive cases at clinically relevant cutoffs [62] (Figure 1). Another key issue is that each validated assay must be performed onto a dedicated autostainer (e.g., 22C3 on AutostainerLink48). However, it is not possible for all laboratories to have all platforms; therefore, in many cases, the PD-L1 IHC tests are performed on different staining platforms as laboratory developed tests (LDT). In this regard, a meta-analysis on the performance of different PD-L1 assays concluded that properly validated LDTs used for the same purpose of a validated PD-L1 assay can perform better than another approved assay that was developed for a different purpose [63]. On this point, we could demonstrate that with an optimized protocol, the non-validated PD-L1 clone E1L3N shows high levels of overall agreement with the SP263 assay in NSCLC [64].

In addition to the complexity related to the different staining performances of the immunohistochemical assays, another issue is represented by the fact that different scoring methods evaluating various cellular compartments within the tumors have been developed. In fact, in clinical trials on pembrolizumab and nivolumab, the predictive potential of PD-L1 expression has been based on its evaluation on tumor cells only using the tumor proportion score (TPS)/tumor cells (TCs), which is defined as the percentage of PD-L1-positive tumor cells (partial or complete) relative to the total number of viable tumor cells [65,66]. On the other hand, in trials assessing atezolizumab, PD-L1 expression has been evaluated on immune cells (ICs) using SP142 as the percentage of tumor area infiltrated by PD-L1-positive immune cells relative to the total tumor area [67,68]. TPS/TC scoring has shown good interobserver agreement, while the IC algorithm is characterized by poor reproducibility among pathologists [69,70]. In other trials evaluating pembrolizumab in a variety of solid tumors, a combined score has been developed, called combined positive score (CPS) and is defined as the percentage of immune cells (lymphocytes and macrophages) and tumor cells relative to the total number of viable tumor cells. For CPS, only a few trials have evaluated the reproducibility of the algorithm, with contrasting results [71,72]. Therefore, further studies assessing the reproducibility of CPS are needed.

Intratumor heterogeneity is another known challenge that can hamper PD-L1 determination and its predictive value. In clinical practice, tumor tissue can be obtained from surgical specimens, core needle biopsies and fine needle aspiration. Moreover, for most patients, only one lesion is sampled, even in the presence of multiple metastases. In this regard, correlative biomarker studies related to immune checkpoint inhibitor trials contain a heterogeneous mix of sample types and sites; therefore, the question on the most appropriate sample for PD-L1 testing remains unanswered. We and others have demonstrated striking topographical PD-L1 expression differences in NSCLC, especially in adenocarcinomas, which is not attributable to morphology alone but likely underpins subclonal evolution [73,74]. In this setting, we found that the best concordance between tissue microarrays cores (used as surrogate of biopsies) and whole sections (used as the reference) varies according to the PD-L1 expression cutoff used and found that three biopsies showed high sensitivity and specificity [75].

Cytology samples often represent the only available diagnostic material for a considerable proportion of metastatic patients but are generally not used in clinical trials for PD-L1 determination. Cell blocks are accepted for PD-L1 TPS scoring since they present a good correlation with histology [74]; on the other hand, in clinical practice, the possibility of having cytologic smears as the only available diagnostic material on which to perform predictive tests occurs in up to 16% of cases. In an exploratory analysis, we compared PD-L1 staining between cytological smears and whole sections from NSCLC cases and found an overall agreement of 90%. Specifically, we found absolute concordance between smears with PD-L1 expressed in <10% and ≥50% of cells and whole sections with PD-L1 expressed in <50% and ≥50% of cells, respectively. Therefore, immunocytochemistry on cytological smears can be considered a reliable method for the evaluation of PD-L1 expression at the 50% cutoff when positive cells are <10% or ≥50%. However, for cytological smears showing PD-L1 expression in 10–49% of cells, additional tissue sampling may be necessary [76].

Despite the standardization of technical procedures, a number of studies have demonstrated substantial variations between pathologists in the interpretation of PD-L1 staining, with disagreement in up to 25% of cases at a TPS cutoff of 1% [70,77]. Although the clinical impact of such interobserver variations is unclear, they represent an intrinsic limit of human visual interpretation in providing a quantitative assessment of a biomarker located in a heterogeneous context [78]. To this end, more precise approaches to PD-L1 scoring, irrespective of the cell compartment, might benefit from digital pathology and artificial intelligence. Such approach could also allow a more comprehensive evaluation of the immune contexture with the possibility to quantify tumor-infiltrating lymphocytes in light of their possible inclusion in more powerful predictive models. Clearly, such approach would require important infrastructure updates and validations before implementation in clinical practice.

## 5. Additional and Novel Strategies to Improve Patient Selection: Beyond PD-L1

Although the predictive potential of PD-L1 immunohistochemistry can be significantly ameliorated by reducing confounding variables regarding specimen features (biopsies vs. surgical specimens; primary vs. metastasis), interassay variability, scoring system (TPS vs. CPSvs.IC) and interpathologist reproducibility, a single biomarker is unlikely to predict response to treatment with immune checkpoint inhibitors. Therefore, other variables are being evaluated to improve the efficacy of the PD-1/PD-L1 axis blockade and to identify predictive markers for immune checkpoint inhibitors.

### 5.1. Tumor Mutational Burden

Tumor mutational burden (TMB) is defined as the number of somatic mutations/Mb of tumor genome. Tumors with a large number of somatic gene mutations develop a higher neoantigen-specific T cell response, which results in an increased susceptibility to immunotherapy. Consistent with this concept, a high TMB has been shown to predict response to immune checkpoint inhibitors across a diverse range of cancer types, including NSCLC, melanoma, and bladder cancers [79]. The TMB is a very promising biomarker, but it is limited by cost and technological requirements correlated with the whole-exome sequencing (WES) assay, not routinely available in most pathology laboratories. A large panel of targeted next generation sequencing (NGS) has been implemented in clinical practice, and TMB estimations derived from the two methods are highly correlated supporting the use of NGS as a surrogate for WES [80]. On June 2020, the FDA approved the use of pembrolizumab for the treatment of adult and pediatric patients with unresectable or metastatic tumors with a high TMB, defined as ≥ 10 mutations/Mb, that have progressed following prior treatment [81]. However, many challenges remain before the implementation of NGS-based TMB as a predictive biomarker in clinical practice: since there are numerous NGS platforms, this poses a major problem in terms of reproducibility and globally accepted predictive TMB (cut-offs have not been determined). Moreover, TMB may be heterogeneous within the tumor, and high TMB alone does not guarantee response to immune checkpoint inhibitors. To this end, there are several examples of patients with low TMB who responded to immune checkpoint inhibitors, as well as cases of patients with high TMB who failed to respond [82].

### 5.2. Mismatch Repair Deficiency (dMMR)/Microsatellite Instability (MSI)

It has been demonstrated that tumors with mismatch repair (MMR) deficiency respond better to PD-1/PD-L1 inhibitors, since they are more prone to express neoantigens [83]. The DNA MMR system recognizes and corrects insertion, deletion and base pair mismatches that occur during DNA replication. dMMR is primarily caused by the inactivation of one or more of the 4 main proteins: MLH1, MSH2, PMS2 and MSH6. dMMR was first detected in colorectal cancer but can occur in many other tumor types [84]. There are 2 main methods of screening for dMMR: IHC for the 4 MMR proteins MLH1, MSH2, PMS2 and MSH6, and molecular testing to detect MSI.

In May 2017, the FDA granted accelerated approval to pembrolizumab for the treatment of unresectable or metastatic dMMR or MSI-high solid tumors that have progressed following prior treatment, the first cancer site–agnostic approval granted from the FDA [85].

### 5.3. Tumor-Infiltrating Lymphocytes (TILs)

TILs are lymphocytes migrated within tumor stroma or the tumor itself. TIL presence reflects the dynamic phase of the immune system attempt to contrast tumor growth. Clinically, higher TIL numbers have been associated with improved survival in multiple solid tumor types [86,87,88]. Within TILs, cytotoxic CD8^+^ T lymphocytes are the effector cells, and their presence within the tumor may suggest checkpoint inhibitor efficacy. Several studies have evaluated intratumoral CD8^+^ lymphocytes alone or in combination with PD-L1 expression as a predictive biomarker of immunotherapy in different tumors [89,90], showing that PD-L1-expressing tumors lacking an appropriate TIL infiltrate may explain the failure of checkpoint inhibition in a subset of patients. Immunoscore (a standardized immune-based assay that measures intra- and peritumoral T cell infiltration) and multiplex immunohistochemistry, an antibody–protein labeling methodology that allows the simultaneous assessment of multiple proteins of interest on one slide, represent more advanced ways to evaluate TILs [91,92].

## 6. Disruption of PD1/PD-L1 Axis in Cancer Therapy

The first drugs disrupting the PD1/PD-L1 axis approved by the FDA were nivolumab and pembrolizumab for the treatment of advanced melanoma [93,94]. Since then, several trials have been carried out, and several antibodies have been approved in different settings. Indeed, several studies have demonstrated the efficacy of drugs blocking the PD-1/PD-L1 pathway, alone or in combination with other therapies, for the treatment of different adult cancer patients (Table 2). On the contrary, there is a lack of knowledge on the use of anti-PD-1/PD-Ls antibodies towards pediatric tumors, and deeper analyses are still required to evaluate their efficacy in childhood cancer patients. We present studies describing PD-1 and PD-Ls expression in neuroblastoma (NB) as well as the ongoing clinical trials aimed to determine the potential role of both anti-PD-1 and anti-PD-L1 mAbs treatments in the context of hematological and solid pediatric cancers.

### Neuroblastoma and Other Pediatric Tumors

The development of drugs targeting immune checkpoint inhibitors has mainly focused on adult cancers. However, in recent years, there has been a push to evaluate the application of these immunotherapeutic approaches to pediatric tumors. Indeed, PD-L1 expression has been observed in several pediatric hematological tumors, such as Hodgkin lymphoma, diffuse large B cell lymphoma (DLBCL), acute myeloid leukemia, acute lymphoblastic leukemia and glioma [137,138,139,140,141]. Despite the high PD-L1 abundance in hematological malignancies, its expression in pediatric solid tumors is relatively low and variable in different histotypes. Thus, PD-L1 expression was detected in the 72% of NB patients where it was found to correlate with a lower survival rate [142]. In addition, Dondero and colleagues demonstrated that lymphocytes derived from bone marrow samples of metastatic NB patients expressed PD-1, and produced IFN-γ that could in turn induce PD-L1 expression in tumor cells [13]. Considering the potential susceptibility of NB to immunotherapy, different studies have been developed to evaluate the efficacy of PD-1/PD-L1 axis disruption-based therapies. Thus, there are several ongoing clinical trials aimed to determine the potential role of both anti-PD-1 and anti-PD-L1 mAbs treatments. A phase I trial is also analyzing a combination therapy with anti-GD2, 131-l Metaiodobenzylguanidine (mlBG) and Nivolumab in treating patients with relapsed and refractory NB (NCT02914405) [143]. In addition, a recent multicentric, phase 1–2 trial was performed using Nivolumab, with or without ipilimumab combination (NCT02304458) in pediatric patients with different tumors, including rhabdomyosarcoma, Ewing sarcoma, osteosarcoma, neuroblastoma, Hodgkin lymphoma, non-Hodgkin lymphoma and melanoma diseases. The results indicated that Nivolumab was safe and well tolerated in children and young adults and showed clinical benefits in lymphoma, while no significant single-agent activity was observed in the other pediatric solid tumors. Importantly, however, this study defines the recommended phase 2 dose and establishes a Nivolumab safety profile for children, which can serve as the basis for its potential study in combination regimens for childhood cancers [144,145]. In this context, an actively recruiting Phase II clinical trial is evaluating the efficacy of Nivolumab in combination with ipilimumab in children with high-grade primary central nervous system (CNS) malignancies (NCT03130959) [141]. The efficacy of Nivolumab in combination with lirilumab, a human monoclonal antibody that binds the KIR2DL1/2L3 receptor, is under investigation in refractory and recurrent malignancies (NCT02813135) [143]. Moreover, a trial that began in 2018 is evaluating the role of Nivolumab in combination with chemotherapy for the treatment of solid tumors and lymphoma (NCT03585465).

Pembrolizumab was approved by the FDA in 2017 for the treatment of children with refractory Hodgkin lymphoma or patients with relapsed tumors [145]. An ongoing clinical trial is investigating the use of Pembrolizumab in children with PD-L1-positive advanced, relapsed or refractory solid tumors or lymphoma or with advanced melanoma (NCT02332668).

## 7. Concluding Remarks

In recent years, immunotherapy with antibodies disrupting the PD-1/PD-L1 axis revealed an unprecedented breakthrough in the treatment of different tumors. This resulted in a true revolution in the cure of highly aggressive cancers. However, despite this progress, a large fraction of patients do not benefit from the use of ICIs. As discussed in this contribution, further improvements may be reached by a more accurate patient selection. This may be based not only on the standardization of available reagents and methods, such as PD-L1 expression and frequency of TILs, but also on the evaluation of other inhibitory checkpoints which may impair antitumor immune responses, independent of PD-1/PD-L1 interactions. Along this line, other major mechanisms play an important role in dampening immune responses. Thus, different cell types, present in the tumor microenvironment, exploit an array of immunosuppressive mechanisms, which “freeze” effector cells, thus hampering their antitumor activity. For example, myeloid-derived suppressor cells (MDSCs), a heterogeneous immature myeloid population, are able to interact with immune cells, compromising their effector function. Of note, the PD1/PD-L1 axis is among the know mechanisms adopted by MDSCs to suppress the immune effector function of T and NK cells. In particular, the surface expression of PD-L1 correlates with the impairment of tumor-infiltrating lymphocytes (TILs) and with tumor progression/prognosis. In TME, tumor cells and their soluble mediators can induce PD-L1 expression on tumor-infiltrating MDSCs [146,147]. Thus, PD-L1 expressed by MDSCs can suppress NK and T cell activity. In different tumor types, increased PD-L1 + MDSC has been detected and, in some instances, a correlation between the percentage of PD-L1 + MDSC and disease stages or clinical outcome has been reported [148]. In addition, Nitric Oxide (NO) produced by MDSCs has a potent inhibitory effect on NK cells by impairing the Fc receptor-mediated killing, the secretion of IFN-γ, TNF-α and Granzyme B, as detected in MDSC-co-cultured NK cells [149,150]. Other important cells, which favor tumor growth and inhibit antitumor immune responses, are the polarized M2 macrophages, which act both directly by secreting different inhibitory factors and cytokines, and indirectly by inducing suppressive cells and favoring fibrotic reactions [151]. Recognition of the prevalent mechanism(s) operating in given tumors may allow the selective removal of the inhibitory effect, either by specifically targeting suppressive cells (e.g., MDSCs or M2 macrophages) or their soluble products. Thus, the use of combination therapies suitable for given tumors appear to be particularly promising. Of note, novel cell-based adoptive therapies (e.g., CAR-T or CAR-NK cells), although very promising, may encounter similar hurdles, including the PD-1 (or other checkpoint) expression on the engineered cells themselves and the suppressive TME which would render their antitumor activity inefficient. Thus, improvement/standardization of available and new diagnostic approaches allowing the identification of the major suppressive mechanisms in a given tumor may lead to more effective, patient-tailored, tumor therapies.

## Figures and Tables

**Table 1:** Evidence reporting PD-1 expression on human T, NK and ILC cells in different cancers. For each data tumor type, cell subset and reference have been reported. PB: peripheral blood; PE: Plural effusion; TILs: tumor-infiltrating lymphocytes; DLBCL: diffuse large B-cell lymphoma; ADC: Adenocarcinoma; NSCLC: non small cell lung cancer; SCC: squamous cell carcinoma.

Type of Tumors	Cell Subsets	References
Breast Cancer (BC)		
Invasive ductal BC	CD4^+^ TILs	[26]
Primary BC	CD8^+^ TILs	[35]
Melanoma		
Metastatic melanoma lesions	CD4^+^, CD8^+^ TILs	[12]
Metastatic melanoma	CD8^+^ TILs	[23]
Malignant melanoma (MM)	CD8+ TILs	[27]
Follicular lymphoma (FL)	CD4^+^ TILs	[30]
Hodgkin Lymphoma (HL)		
Primary classical HL	CD4+ TILs	[29]
HL and DLBCL	NK PB and Intratumoural	[39]
Ovarian carcinoma	NK peritoneal fluid/ascites	[7]
Karposi sarcoma	NK PB	[38]
Renal cell carcinoma (RCC)	NK PB	[40]
Lung cancers		
Primary and metastatic	ILC3 PE	[44]
Lung cancer and ADC	NK PE	[47]
	NK PB	[42]
NSCLC (ADC and SCC)	CD8^+^ TILs	[28]
Advanced and primary NSCLC	CD8^+^, CD4^+^ TILs	[27,32]
NSCLC	NK Intratumoural	[43]
Digestive Cancers		
Gastric cancer	CD8^+^ TILs	[27]
Gastrointestinal (oesophageal, gastric, colon, rectal tumors)	ILC2, ILC3, NK intratumoral	[45]
Hepatocellular carcinoma (HCC)	CD8^+^ TILs	[25]
	CD4^+^, CD8^+^ TILs	[33]
ESCC, HCC, colorectal cancer and biliary cancer	NK Intratumoural and PB	[37]
Oesophageal cancer	CD4^+^ CD8^+^ PB and TILs	[34]
Multiple Myeloma	NK PB	[36]

**Table 2:** Current indications for anti PD1/PD-L1 therapy in different advanced/metastatic tumor types: NSCLC: non-small cell lung carcinoma; SCLC: small cell lung carcinoma; TNBC: triple negative breast carcinoma; CSCC: cutaneous squamous cell carcinoma; RCC: renal cell carcinoma; UC: urothelial carcinoma; HCC: hepatocellular carcinoma; GEJ: gastro-esophageal junction; MSI: microsatellite instability; dMMR: mismatch repair deficient; HNSCC: head and neck squamous cell carcinoma; HL: Hodgkin lymphoma; PMLBCL: primary mediastinal large B cell lymphoma; wt: wild-type; TPS: tumor proportion score; CPS: combined positive score; IC: tumor-infiltrating immune cell; pembrolizumab, nivolumab, cemiplimab: anti-PD-1; atezolizumab, durvalumab, avelumab: anti-PD-L1.

Type	Treatment	Indications (Ref)
NSCLC	Pembrolizumab	I line (*ALK* and *EGFR* wt, TPS ≥ 1%) [95] II line (TPS ≥ 1%) [96]
	Pembrolizumab + chemotherapy	I line [97,98]
	Atezolizumab	I line (*ALK* and *EGFR* wt, TPS ≥ 50% and/or IC ≥ 10%) [99] II line [100]
	Atezolizumab + chemotherapy	I line in non-squamous histology, *ALK* and *EGFR* wt [101]
	Nivolumab	II line [102]
	Durvalumab	After chemoradiation for unresectable stage III NSCLC [103]
SCLC	Atezolizumab + chemotherapy	I line [104]
	Durvalumab + chemotherapy	I line [105]
	Nivolumab	II line [106]
TNBC	Atezolizumab + chemotherapy	I line (IC ≥ 1%) [107]
Melanoma	Pembrolizumab, Nivolumab	Adjuvant treatment after radical surgery [108,109]; I line [110,111]; II line [93,112]
Merkel cell carcinoma	Pembrolizumab	I line [113]
	Avelumab	II line [114]
CSCC	Cemiplimab	I line [115]
RCC	Nivolumab + ipilimumab	I-II line [116,117]
	Pembrolizumab/avelumab + axitinib	I line [118,119]
UC	Pembrolizumab	I line in patients ineligible for cisplatin-containing therapy (CPS ≥ 10%), or patients unfit for platinum-containing chemotherapy [120]
	Atezolizumab	I line in patients ineligible for cisplatin-containing therapy (IC ≥ 5%), or patients unfit for platinum-containing chemotherapy [121]
	Atezolizumab, Nivolumab, Durvalumab, Avelumab, Pembrolizumab	Disease progression during or after platinum-based chemotherapy or within one year after adjuvant or neoadjuvant chemotherapy [120,122,123,124,125]
Cervical cancer	Pembrolizumab	II line (CPS ≥ 1%) [126]
HCC	Nivolumab/Pembrolizumab	II line [127,128]
Esophageal cancer	Pembrolizumab	II line (CPS ≥ 10%) [129]
Gastric/GEJ adenocarcinoma	Pembrolizumab	II line (CPS ≥ 1%) [130]
MSI-H dMMR cancers	Pembrolizumab	II line irrespective of primary location [131]
HNSCC	Pembrolizumab	I line (CPS ≥ 1%) [132]
	Pembrolizumab/Nivolumab	II line [133,134]
HL	Pembrolizumab	II line [135]
PMLBCL	Pembrolizumab	II line [136]
